# Surgical treatment of lumbosacral tuberculosis by one-stage debridement and anterior instrumentation with allograft through an extraperitoneal anterior approach

**DOI:** 10.1186/s13018-015-0204-x

**Published:** 2015-05-10

**Authors:** Jian-Hua Li, Ze-Hua Zhang, Tao Shi, Fei Dai, Qiang Zhou, Fei Luo, Tian-Yong Hou, Qing-Yi He, Mo-Yuan Deng, Jian-Zhong Xu

**Affiliations:** Department of Orthopaedics, Southwest Hospital, Third Military Medical University, Gaotanyan Street 29, Shapingba District, Chongqing, 400038 China; National & Regional United Engineering Laboratory of Tissue Engineering, Southwest Hospital, Third Military Medical University, Chongqing, China

**Keywords:** Anterior approach, Tuberculosis of lumbosacral junction, Internal fixation

## Abstract

**Background:**

This study was aimed to investigate the clinical outcome of lumbosacral tuberculosis treatment by one-stage radical debridement with bone allograft reconstruction and anterior instrumentation via a retroperitoneal approach.

**Methods:**

We retrospectively analyzed a series of 43 patients with lumbosacral tuberculosis in whom the lumbosacral junction was exposed via an anterior midline retroperitoneal approach. After radical debridement, two parallel tricortical iliac crest bone allografts were placed to reconstruct the anterior column, and then anterior fixation was performed.

**Results:**

The mean follow-up period was 34 months (range, 24–91 months), during which no obvious loss of correction was observed. No case experienced recurrence, tuberculous peritonitis, erectile dysfunction, or retrograde ejaculation.

**Conclusions:**

The midline retroperitoneal approach provides direct and safe access to lesions of lumbosacral tuberculosis. Two parallel structural iliac crest allografts and anterior instrumentation effectively stabilize the lumbosacral junction.

## Background

Lumbosacral tuberculosis is a rare condition, representing only 2%–3% of all cases of spinal tuberculosis [1]. Surgical stabilization of the spinopelvic region in patients with lumbosacral tuberculosis remains a challenge clinically due to the complex anatomy and biomechanics of the affected region. Most of the previously reported procedures adopt the single- or two-stage combined anteroposterior approaches. Despite good clinical outcomes, the surgical trauma involved in these procedures is severe and the cost is relatively high [2]. In addition, the anterior surgical approach allows for direct and complete removal of the lesion, relief of spinal cord compression, and restoration/recovery of spinal stability. It has been shown that one-stage decompression, fusion, and internal fixation can achieve excellent outcomes in the treatment of thoracolumbar tuberculosis [3-5]. From July 2001 to December 2011, we treated 43 patients with tuberculosis of the lumbosacral junction with a one-stage operation involving lumbar interbody fusion with allografts via the retroperitoneal approach.

## Methods

### Clinical data

In this retrospective study, we analyzed outcomes for all 43 consecutive patients treated for lumbosacral tuberculosis at our hospital from July 2001 to December 2011. Patients included 20 males and 23 females with a median age of 40 years (range, 20–69 years). The disease duration prior to treatment ranged from 1 month to 6 years (mean, 15.47 months). Among these patients, 27 had complained severe or moderate back pain; 21 reported radiation pain in the lower limb; and 16 had muscle strength loss, pain, and hypoesthesia in the lower limbs. Plain radiography, computed tomography (CT), and/or magnetic resonance imaging (MRI) were performed on all the patients before surgery and showed that the lesions were all located in the L5–S1 segments. Paravertebral abscesses were observed in 40 patients, and vertebral damage was clearly observed in all patients. Intervertebral space was found to be narrower. Overall, 26 cases presented with presacral abscess, 9 cases with fistula from L5 paravertebral abscesses, 16 cases with pulmonary tuberculosis, and 7 cases with sacroiliac joint tuberculosis. Before surgery, the average lumbosacral angle was 13.30° (range, 5°–20°) [6,7], and the average blood sedimentation rate was 45.67 mm/h (range, 25–76 mm/h). Antituberculosis chemotherapy was administered for at least 2 weeks (Tables 1 and 2).

**Table 1:** **Patient demographics, operative information, and disease characteristics**

**Patient**	**Age (years)**	**Gender**	**Operation time (minutes)**	**Blood loss (mL)**	**Level**	**Duration of symptoms (months)**	**Length of follow-up (months)**
1	25	M	145	200	L5–S1	18	27
2	23	M	150	200	L5–S1	12	27
3	53	F	150	200	L5–S1	6	33
4	20	M	170	150	L5–S1	36	27
5	50	M	170	150	L5–S1	12	27
6	36	F	285	500	L5–S1	12	91
7	25	M	210	200	L5–S1	12	28
8	33	F	175	200	L5–S1	12	30
9	46	F	160	250	L5–S1	7	32
10	61	F	525	600	L5–S1	36	73
11	59	M	645	800	L5–S1	4	51
12	26	F	755	800	L5–S1	24	50
13	41	M	180	350	L5–S1	12	33
14	30	F	765	800	L5–S1	60	60
15	65	M	540	400	L5–S1	1	27
16	47	M	348	400	L5–S1	3	30
17	69	M	80	200	L5–S1	5	33
18	54	M	172	200	L5–S1	3	43
19	40	F	167	200	L5–S1	6	33
20	57	M	190	300	L5–S1	5	33
21	35	F	125	250	L5–S1	6	39
22	37	F	160	600	L1–S1	60	27
23	25	M	500	1600	L2–S1	2	27
24	40	F	135	200	L5–S1	7	35
25	60	F	210	500	L5–S2	6	29
26	53	M	100	150	L5–S1	12	39
27	24	M	177	200	L3–S1	24	28
28	23	M	132	200	L5–S1	24	32
29	47	F	151	200	L5–S1	72	27
30	44	M	188	400	L4–S2	12	27
31	68	F	148	400	L5–S2	2	33
32	31	F	159	800	L4–S1	10	51
33	21	M	170	800	L5–S1	12	27
34	39	F	127	300	L5–S1	12	27
35	44	M	147	200	L5–S1	12	31
36	24	M	156	200	L5–S1	4	29
37	25	M	225	300	L5–S1	9	28
38	44	M	112	200	L5–S1	8	27
39	36	M	241	600	L5–S1	4	26
40	46	F	208	500	L5–S1	24	25
41	33	F	110	200	L5–S1	3	25
42	36	F	140	300	L5–S1	24	43
43	34	F	130	300	L5–S1	30	24
Mean ± SD	40.21 ± 13.75	-	231.00 ± 170.75	383.72 ± 280.46		15.47 ± 16.18	34.74 ± 13.45

*SD* standard deviation, *M* male, *F* female.

**Table 2:** **Radiological and clinical outcomes**

**Patient**	**Lumbosacral angle (°)**			**VAS score**			**ESR (mm/h)**			**Bone fusion (months)**
	**Preop**	**Postop** ^**a**^	**Final**	**Preop**	**Postop** ^**b**^	**Final**	**Preop**	**Postop** ^**a**^	**Final**	
1	7	16	14	7	1	0	47	10	12	9
2	10	15	14	7	1	0	60	26	7	8
3	15	22	20	8	2	0	58	26	11	10
4	5	17	15	5	1	1	39	20	7	8
5	20	29	28	9	1	1	46	13	9	8
6	13	24	22	7	2	1	54	11	9	9
7	16	28	24	7	1	1	31	5	6	9
8	20	30	28	8	2	0	29	5	8	9
9	11	13	17	7	1	0	31	5	8	8
10	16	25	24	9	2	1	25	6	9	8
11	11	13	12	7	1	0	25	6	11	9
12	15	22	21	8	2	0	29	14	11	9
13	20	27	26	8	2	1	46	7	11	9
14	17	26	25	8	2	0	57	11	8	8
15	8	18	14	6	1	0	33	6	9	9
16	11	16	15	7	1	0	59	18	7	8
17	12	23	21	7	2	1	76	11	7	9
18	12	23	22	7	2	0	39	17	14	10
19	9	17	17	6	1	0	35	11	9	9
20	20	30	28	8	2	0	62	12	8	8
21	16	28	23	7	1	1	66	14	11	8
22	12	23	23	7	2	1	30	13	11	9
23	11	13	16	7	1	0	42	8	9	8
24	16	25	25	9	2	1	47	7	7	11
25	13	24	22	7	2	1	50	16	11	9
26	11	13	14	7	1	0	29	13	12	9
27	11	13	14	7	1	0	39	12	8	9
28	6	17	15	7	2	1	46	7	9	9
29	9	19	17	6	1	1	62	12	8	9
30	12	21	21	7	2	0	45	14	9	8
31	20	27	25	9	1	1	53	15	7	8
32	18	26	24	9	1	1	61	10	12	8
33	17	25	23	9	1	1	37	9	10	8
34	15	25	24	8	2	0	64	16	11	8
35	10	15	13	6	1	1	74	14	11	9
36	9	15	13	6	1	1	48	9	9	8
37	13	24	21	7	2	0	32	12	7	8
38	14	27	24	7	2	1	36	14	11	8
39	19	25	25	8	2	0	55	8	9	9
40	14	25	24	8	2	0	55	4	8	9
41	11	16	15	7	1	0	31	7	8	10
42	13	25	23	7	2	1	34	9	7	10
43	14	24	20	8	2	0	47	9	10	10
Mean ± SD	13.3 ± 3.96	21.6 ± 5.33*	20.26 ± 4.78*	7.35 ± 0.95	1.51 ± 0.51*	0.47 ± 0.50*	45.67 ± 13.56	11.44 ± 5.04*	9.21 ± 1.83*	8.74 ± 0.76

*ESR* erythrocyte sedimentation rate, *preop* preoperative, *postop* postoperative, *SD* standard deviation, *VAS* visual analogue scale.

*Indicates a statistically significant difference between the mean postop or final value and the mean preoperative value (all *P* < 0.01).

^a^Postoperative follow-up at 3 months.

^b^Postoperative follow-up at 6 months.

### Surgical procedure

**Figure 1:**
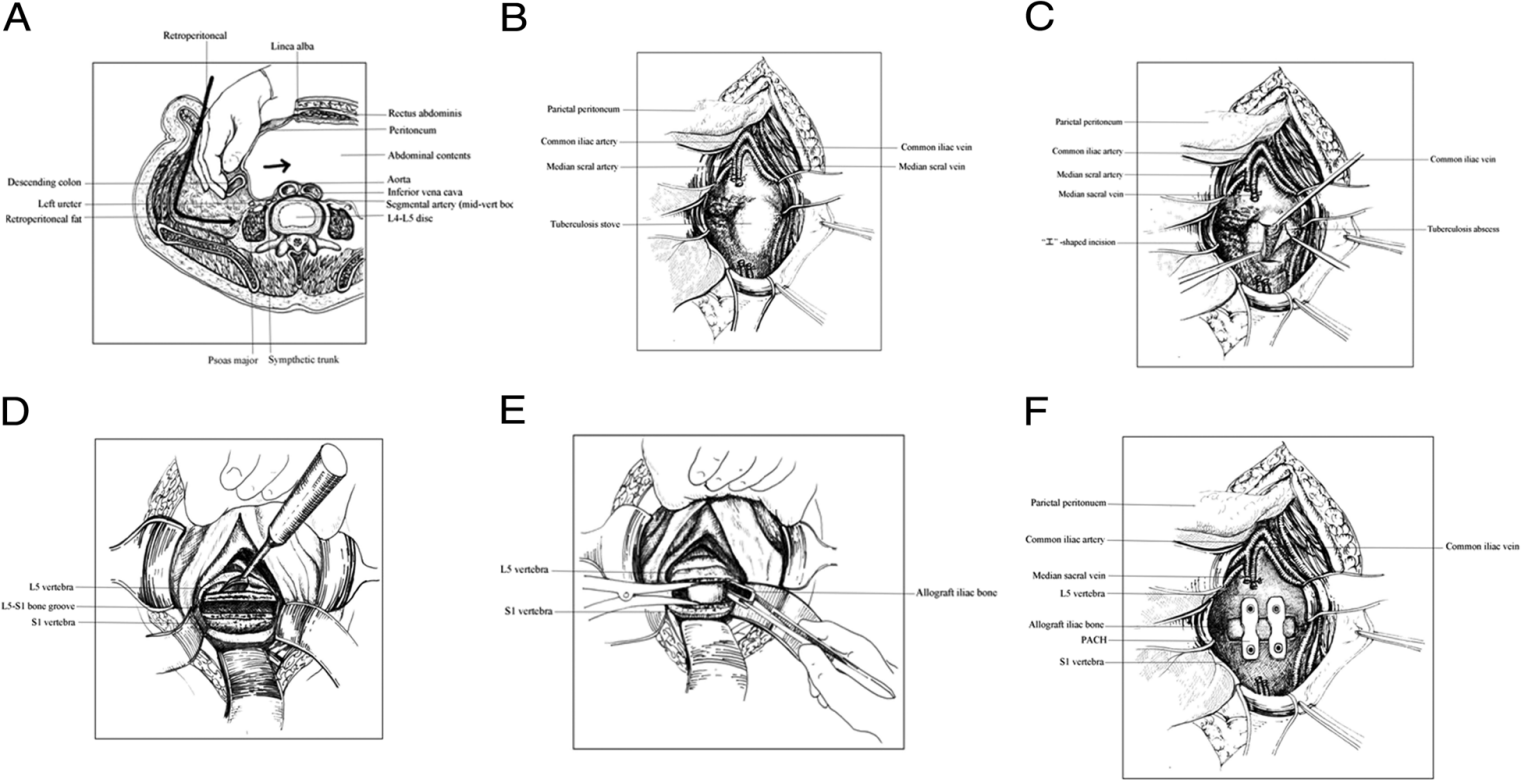
The surgical procedure used in the study. **(A)** Exposure of the anterior lumbosacral spine. **(B)** Ligation of the median sacral artery and vein. **(C)** An “H”-shaped longitudinal incision and removal of pus. **(D)** The pus, caseous necrotic tissue, granulation tissue, necrotic bone, and intervertebral disc are removed. **(E)** Two tricortical allograft iliac bones were trimmed to the proper size and then tightly inserted into the L5–S1 bone groove. **(F)** Two self-locking titanium anterior lumbosacral vertebrae plates (PACH; General Corp., Germany) of suitable length were selected and anteriorly fixed at L5–S1.

The patient was placed in the supine position with the feet higher than the head. Epidural anesthesia was induced, and then a median incision was made through the skin along the symphysis pubis to the umbilicus. The skin and subcutaneous tissue were dissected off the anterior rectus sheath. The parietal peritoneum was bluntly dissected using the hand and gauze, and the peritoneum and its contents were slightly pushed inward to expose the anterior lumbosacral spine (Figure 1A). Then the sacral promontory was located, and the abdominal aorta, inferior vena cava, and its bifurcations were identified. The median sacral artery and vein were ligated, and then a horizontal H-shaped longitudinal incision was made along the midline with the sacral promontory as the center to reveal the lesions, during which the hypogastric plexus and the ureters were carefully protected. The prevertebral fascia and the abscess were cut open to drain the pus (Figure 1B, C). The lesion was isolated with wet gauze to prevent contamination of the surrounding tissues and spread of *Mycobacterium tuberculosis*. A curette and rongeur were used to clear the pus, caseous necrotic tissue, granulation tissue, necrotic bone, and intervertebral disc (Figure 1D). The wound was irrigated with hydrogen peroxide and then physiological saline repeatedly until the effluent was clear. The intervertebral space was expanded appropriately to measure the extent of the bone defect. Two tricortical allograft iliac bones were trimmed to the proper size and then tightly inserted into the L5–S1 bone groove (Figure 1E). Two self-locking titanium anterior lumbosacral vertebrae plates (PACH; General Corp., Germany) of suitable length were selected and anteriorly fixed at L5–S1 (Figure 1F). Hemostasis was achieved, and the wound was thoroughly irrigated. Then 1 g of streptomycin and 0.6 g of isoniazid were placed around the implant [8]. Then, the normal cancellous bone collected during the procedure outlined in Figure 1D and demineralized bone matrix (DBM) were mixed and implanted into the gap around the steel plates on both sides of the grafted bone. For patients with sacroiliac joint tuberculosis, in which the abscess was in front of the joint, the surgical techniques can be similar to the procedure described, and the surgical procedure may differ in other conditions. The incision was sutured layer by layer. The stitches were removed 10 days after surgery after the wounds had healed well. A thoracolumbar orthosis was maintained for 6 months after surgery, combined with antituberculous chemotherapy for 12 months.

### Surgical evaluation

During the surgery, the abdominal aorta, inferior vena cava, and its bifurcations were identified as the first key points, and the hypogastric plexus and ureters were carefully protected during the process of clearing the pus. The pus must be cleared completely, and the nerves, blood vessels, and other organs not be hurt. The two tricortical allograft iliac bones must be tightly inserted into the L5–S1 bone groove, and the PACH should be impartial. We could check them and could also see the effect by X-ray. After the surgery, it was necessary to pay attention to the basic state of the patient, checking the ESR, CRP, etc. X-ray and CT were repeated at 3 days after the surgery. If the patient had no serious hemorrhoids or defecatory, urinary, or erectile dysfunction and the device was not displaced, we considered the surgery to be successful. All patients were followed up systematically, and outcomes were analyzed. The Bridwell criteria were used to estimate the degree of bone union in the patients.

## Results

The mean operative time was 231.00 min (range, 80–765 min), and the mean blood loss volume was 383.72 mL (range, 150–1600 mL). No injuries to the large blood vessels, nerves, or ureters occurred in these patients, and none of the 20 male patients experienced erectile dysfunction during the follow-up period. None of the patients developed wound infection or fistula. Two patients suffered abdominal distention, and two patients suffered urinary retention after surgery. The 43 patients were followed up for a mean period of 34.74 months (range, 24–91 months), and no patients experienced recurrence of the disease during follow-up. It should be mentioned that the recurrence rate is 2%–5% in the long term (5–10 years) [9-11].

**Figure 2:**
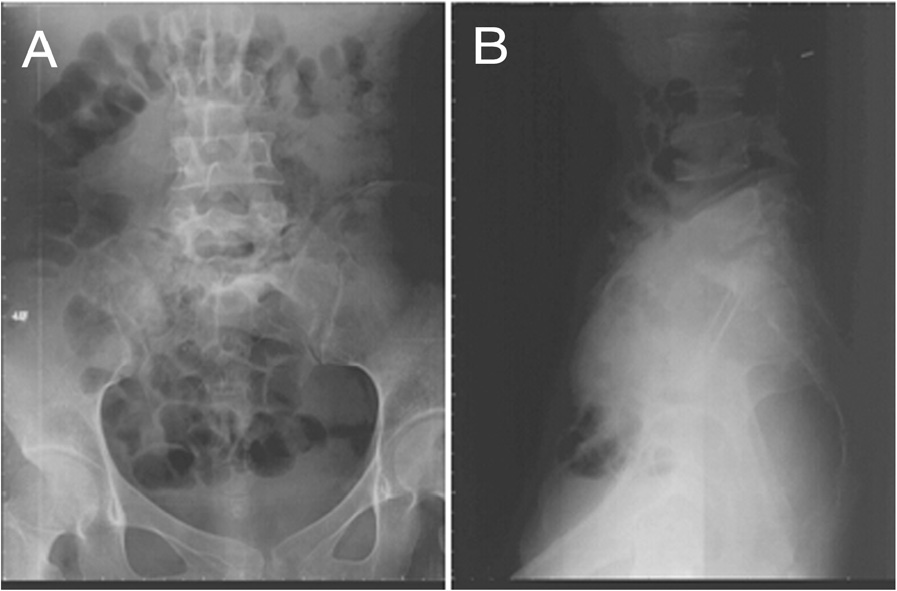
X-ray showing L5–S1 vertebral damage with narrowed intervertebral space and lumbosacral angle of 16°. **(A)** Anteroposterior view and **(B)** lateral view.

**Figure 3:**
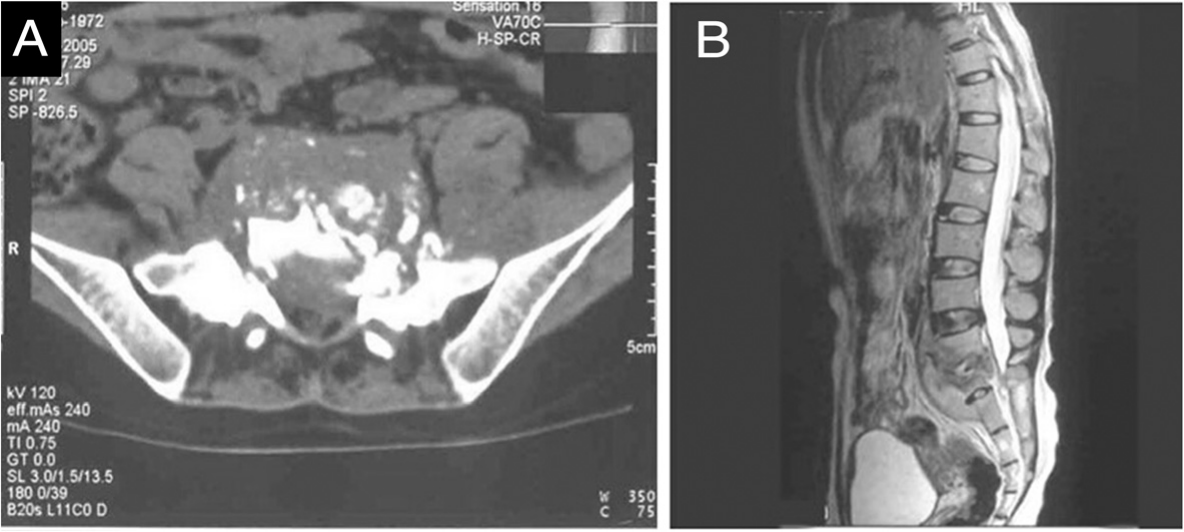
CT and MR images showing bone union of grade I. **(A)** CT image showing S1 bone destruction, presacral cold abscess, and sequestrum. **(B)** MRI showing L5–S1 vertebral signal change, narrowed intervertebral space, and presacral cold abscess.

**Figure 4:**
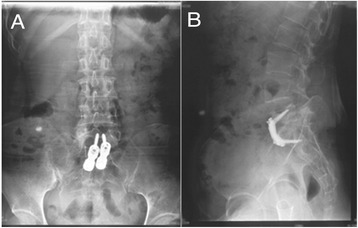
At 1 year postoperatively, the lumbosacral angle was 23°. **(A)** Anteroposterior view and **(B)** lateral view.

**Figure 5:**
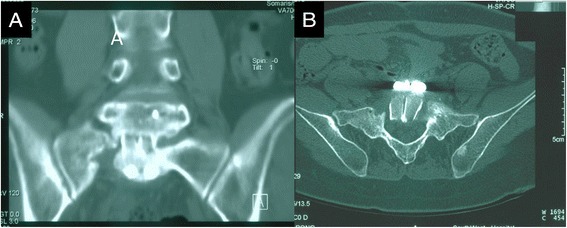
One year after operation, CT images showed that the trabecular bone was reconstructed well without photic zone, grade I bony fusion. **(A)** Anteroposterior view and **(B)** axial view.

Partial absorption of the edge of the bone was observed in two patients. According to the Bridwell criteria [12], at the last follow-up, bone union of grade I (Figures 2,3,4, and 5) occurred in 41 cases, with the healing time ranging from 8 to 11 months, and bone union of grade II occurred in 2 cases 11 months after the surgery. In addition, the erythrocyte sedimentation rate (ESR) was found to be normal, and back pain with radiation in the lower limbs had disappeared. The mean lumbosacral angle was 21.60° (range, 13°–30°) after the operation and 20.26° (range, 13°–28°) at the last follow-up (Tables 1 and 2).

## Discussion

L5–S1 is a spinal segment located between the lumbar lordosis and sacral kyphosis. As these vertebrae bear the majority of body weight, the stability of this segment is critical. In addition, the upper surface of S1 vertebra slants forward, and therefore, there is a high risk of L5 spondylolisthesis. The L5–S1 zygopophysis and intervertebral disc are the main structures responsible for resisting L5 spondylolisthesis. In patients with L5–S1 vertebral tuberculosis, the damaged intervertebral discs and vertebrae, as well as the decreased lumbosacral angle, may dislocate the L5–S1 joint, leading to joint capsule laxity. Therefore, the L5–S1 intervertebral disc must be removed during debridement of tuberculosis lesions. However, without the intervertebral disc, the shear stress between L5 and S1 can cause accelerated degradation of the zygopophysis, leading to back pain. Thus, an important goal of treatment is to recover the height of vertebrae and prevent local shear and axial stresses via bone graft fusion. However, the intervertebral bone graft may slip forward during rotation or extension movement of the lumbar spine, and it is essential to use internal fixation for early reconstruction and maintenance of the vertebrae [13-16]. A posterior approach to fixation of the lumbosacral segments has been performed at a safe distance from the tuberculosis lesion but is time-consuming and quite invasive [2]. Here, we tested the effectiveness and safety of one-stage radical debridement with bone allograft reconstruction and anterior instrumentation via a retroperitoneal approach to treatment patients with lumbosacral tuberculosis.

The anterior approach to the lower lumbar spine includes the extraperitoneal or intraperitoneal approaches. The common iliac artery and vein are located anterior-laterally to the L5 vertebra. Although the extraperitoneal approach can clearly expose the lumbosacral segments, this approach can lead to injury of the blood vessels and ureters. On the contrary, the intraperitoneal approach to the L5–S1 anterior interbody fusion has the following advantages: (1) incision through the linea alba can avoid muscular damage; (2) lesions located anteriorly and bilaterally to the sacrum can be fully exposed and debrided, which can facilitate the anterior internal fixation; and (3) a direct view can minimize surgical damage to blood vessels, nerves, and ureters [17,18]. In our patients, only mild complications occurred postoperatively, such as abdominal distention and urinary retention, which resolved spontaneously without special treatment. No severe complications, such as tuberculous peritonitis, intestinal obstruction, erectile dysfunction, and retrograde ejaculation, were observed during the long-term follow-up.

Most patients with spinal tuberculosis will have good outcomes after conservative treatments. However, the surgical approach applied in this study may be considered in patients with the following conditions: large presacral abscess or sequestrum, severely damaged vertebrae that require reconstruction for stability, history of previous multiple surgeries conducted via the transperitoneal approach with local tissue and intestine adhesion, and female.

Through 100 years of development, allograft bone transplantation has become a mature technology with minimal risks of immune rejection and infection [19-21]. The American Association of Tissue Banks (AATB) has proposed and continually updates guidelines for the acquisition, processing, sterilization, and preservation of allogeneic bones, and most bone banks worldwide adopt these recommendations [22]. Allograft bone transplantation has been used for lumbar interbody fusion in the treatment of spinal tuberculosis and achieved good outcomes. Most of these allografts are obtained from freshly frozen femur or humerus rings [23-29]. In our patients, freeze-dried iliac allografts were used for lumbar interbody fusion. We found that the allogeneic iliac blocks can easily be trimmed to the desired shapes and sizes. The tricortical iliac crest bone grafts also have good biomechanical performance and can withstand strong compression forces. The cancellous bones at both ends of the grafts immediately contact the cortical bones of the upper and lower vertebrae, facilitating the replacement by host bone tissues. In this study, we inserted two allogeneic iliac crest bone blocks in a parallel manner into the L5–S1 intervertebral space and achieved good spine stability.

Postoperative radiographs and CT images at 3–6 months showed partial absorption of the upper or lower edge of the allografts in two patients. This may have been associated with the replacement of the allogeneic bones and slight loosening of the internal fixation. The two patients achieved grade II bone union at postoperative 9 and 11 months, respectively. The time to bone union in these patients was obviously longer than that achieved with the use of autografts. However, the allografts were satisfactory in terms of deformity correction and stability restoration. In addition, allogeneic iliac crest bone grafting has advantages over autografts including shorter operation time and avoidance of donor site morbidity.

## Conclusions

In conclusion, the midline retroperitoneal approach provides direct and safe access to lesions of lumbosacral tuberculosis. Moreover, interbody fusion with iliac crest allografts and anterior instrumentation can effectively restore the lumbosacral junction stability.

## Abbreviations

CT: Computed tomography
MRI: Magnetic resonance imaging
DBM: Demineralized bone matrix
